# The emerging trends of healthcare professions’ ethics education in recent 10 years: a systematic review

**DOI:** 10.3389/fmed.2026.1748761

**Published:** 2026-03-17

**Authors:** Xiyang Yin, Wei Wei, Yinjia Zheng

**Affiliations:** 1 Faculty of Applied Sciences, Macao Polytechnic University, Rua de Luis Gonzaga Gomes, Macao, China; 2 Department of Teaching Management, The Fifth Affiliated Hospital of Guangzhou Medical University, Guangzhou, Guangdong, China

**Keywords:** competency-based programs, ethics education, healthcare professions, mutual safety, professionalism, virtues and character

## Abstract

**Background:**

Because of globalization and advancement of AI technology, the trends of healthcare professions’ ethics education have changed significantly. However, the available literature reviews may not reflect current trends, lacking consistency in the teaching topics, and assessment standards of ethics education. Furthermore, no systematic review has examined the relationship between different medical ethics education topics.

**Objective:**

To fill these gaps, it is a key point that medical educators and institutions work toward establishing a standardized and comprehensive framework for medical ethics education. In this case, this research aimed to provide a comprehensive review of the academic research surrounding medical ethics education for medical students and professions.

**Methods:**

A systematic search following PRISMA guidelines conducted across databases, including PubMed and Web of Science for recent papers published on medical ethics education from 2015 to 2025. Post-screening, 145 studies met the final criteria.

**Results:**

145 studies were included in the current review. Four core themes were identified: virtues-based ethics education, competency-based ethics education, professionalism, and mutual safety. Results indicated that competency-based ethics education was the area most frequently discussed in the publications (*N* = 81, 55.86%). There were interrelationships exist among the four themes, particularly between virtues-based ethics education and professionalism (*N* = 18, 12.41%). This study also introduced a novel integrative framework and emphasized the new role of mutual safety, which can enhance healthcare professions’ ethics and clinical practice.

**Conclusion:**

Medical ethics education plays an important role in clinical practice and moral decision-making. This review highlights the need for a standardized, multidimensional approach to understand the emerging trends of healthcare professions’ ethics education, which will be helpful for AI education in medical ethics education in the future.

## 1 Introduction

Medical ethics is an intellectual discipline (1), which is a broad field that links the healthcare setting with ethical concerns. In modern society, medical ethics is still a clinical system of institutionalized moral principles and procedures that helped shape new modes of medical practice (2). Although there are ongoing efforts to instill transferable ethical knowledge, moral skills and professionalism in medical students, the effectiveness of existing ethics curricula in achieving this goal is now under scrutiny (3). In fact, researchers found that medical students often feel underprepared to handle ethical challenges during clinical internships (4). Additionally, moral distress, burnout, and attrition among practicing physicians have increased significantly (5), eroding their professionalism (6). It seems that there is a gap between their education and practical demands.

Despite this widespread adoption of ethics education in medical school and the publications of several systematic reviews on this field (2, 4, 7), three main questions remain about the medical ethics education. Firstly, most present systematic reviews focused on medical students in ethics learning (2, 4, 8, 9). However, they did not describe the medical students’ ethical development and changes when they are entering into clinical practice. This question may help us to understand how medical ethics can be most effectively integrated into clinical practice. However, the present reviews only assessed the effectiveness of medical students’ ethics learning (2, 4), while Azim and Shamim (10) focused solely on educational theories. Some reviews also yielded a small number of included articles: 29 (2), 18 (9), 8 (11), and 6 (10). Secondly, the primary goal of medical ethics education has not been uniformly defined across institutions, leading to confusion and inconsistency in curricula. Researchers aimed to explore the similarities in teaching goals and prescribed content, contributing to a more comprehensive understanding of the current state of medical ethics education. According to Jegan and Dierickx (12), they pointed out that medical education and bioethics have undergone a “global turn” in the process of globalization. At the same time, artificial intelligence (AI) technology plays a crucial role in advancing globalization (13), which can create conditions for standardization and unification of ethical education. Thirdly, the interrelationship between the various goals of the emerging trends of healthcare professions’ ethics education remains inadequately explored. Several systematic reviews in medical ethics education have been explored in the past. However, they were generally concerned about one or two topics in medical ethics education. For example, Wan et al. (8) solely focused on virtues-based programs of medical education in medical students. They did not explore whether virtue can influence moral competence or professional skills or not.

To fill these gaps, it is a key point that medical educators and institutions work toward establishing a standardized and comprehensive framework for medical ethics education. Furthermore, no systematic review has examined the relationship between different medical ethics education topics. In this case, this research hopes to provide a comprehensive and current review of the academic research surrounding medical ethics education for medical students and professions. In this systematic review, the authors summarize the deficits in the current literature of medical ethics education, outline a novelty framework on medical ethics education, and reveals the dynamic relationship among the goal of medical ethics education. To summarize, this research involves a review of the studies of healthcare professions’ ethics education. This review focuses on articles published in the period from 2015 to 2025 from the PubMed and Web of Science. The research questions that guided this review are as follows: (1) what are the emerging trends of healthcare professions’ ethics education in recent 10 years; (2) what is the interrelationship between the various goals of the emerging trends of healthcare professions’ ethics education?

## 2 Methods

### 2.1 Protocol

We searched related publications utilizing a three-step process, article search and selection, and data analysis. This review is reported according to the Preferred Reporting items for Systematic Reviews and Meta-Analyses (PRISMA) statement (14).

### 2.2 Focus question

A modification of the PICO (population, intervention, comparison, outcome) framework, PIO (population, intervention, outcome), was employed to design the initial search criteria (15, 16). The focus question was conducted according to the Patient and population, Intervention and Outcome (PIO) framework as described in Table 1.

**TABLE 1:** PIO framework.

Elements	Description
(P) Population	Healthcare professionals’ students, residents, nurses, healthcare professionals or patients’ teachers
(I) Intervention	The exposition, discussion, and conceptualization of healthcare professions’ ethics education
(O) Outcomes	Outline the educational strategies to enhance healthcare professions’ ethics education

### 2.3 Information sources and search

A systematic and comprehensive literature search was conducted in the PubMed database to identify relevant publications. The search strategy was designed to capture studies at the intersection of moral or ethics education and the medical field. The specific search strategy (Query box of Advanced Search Builder) executed in PubMed is detailed below: [(“Morals”(Mesh) OR “Ethics”(Mesh) OR “Bioethical Issues”(Mesh) OR “moral education”(tiab) OR “ethics education”(tiab) OR “ethical reasoning”(tiab)] AND [“Education”(Mesh) OR “Education, Medical”(Mesh) OR “Curriculum”(Mesh) OR teaching (tiab) OR learning (tiab)] AND [“Education, Medical”(Mesh) OR “Physicians”(Mesh) OR “Students, Medical”(Mesh) OR medical (tiab) OR medic*(tiab) OR physician*(tiab) OR doctor*(tiab)]. In addition, we applied a database filter: Full text, Introductory Journal Article, Review, Scoping Review, Systematic Review, English, from 2015 to 2025. This review study was also searched in Web of Science Core Collection, “English” as the language. This systematic review focuses on the topic of moral judgment within the field of medicine, specifically examining the intersection of education and psychological disciplines. For quality assurance, the literature search was conducted across articles indexed in the Science Citation Index (SCI) and Social Sciences Citation Index (SSCI) published from 2015 to 2025. The document type was required as “article.” This approach improves the rigor of the research and ensures that the analyzed literature provides deeper empirical support and data analysis. The following search strategy adopted a combination of two topics (topic 1: moral education or ethics education; topic 2: medical). The terms were combined using Boolean statements (moral education or ethics education and medical). Key categories included education scientific disciplines, educational research, psychology in education, and special education (see Supplementary Table 1).

### 2.4 Selection of studies

The present search was conducted from 2015 to the end of April 2025, generating 402 articles in Web of Science Core Collection, and the present search was conducted in 2015 to the end of 2025 generating 281 articles in PubMed. They were further examined based on the following inclusion criteria. Firstly, this review excludes the academic ethical education of healthcare professionals. Secondly, in this study, the focus is on healthcare professionals, medical students or medical ethics teachers, rather than only discussing ethical biotechnology. Thirdly, the publications had to be journal articles that investigated ethics education in medicine. Fourthly, medical ethics education should be the main topic. Magalhães-Sant’Ana (17) approach to thematic analysis and the new concept of mutual safety (18) were used to identify common themes across different themes (example of coding table as shown in Supplementary Table 2). Finally, the 145 articles were included for the systematic review. Studies that met the article selection criteria were submitted to full-text review, which followed the same strategy.

To fill in the research gaps the researchers analyzed the selected literature from three aspects: the research themes, the sub themes, and the main research populations. The two authors initially reviewed 40 articles together to generate initial coding. In case of any discrepancies, they would re-read the literature and discuss the coding scheme until they reached a consensus. Disagreements on four of the papers were resolved through discussion; for one paper where the two initial raters could not agree, the researchers consulted a third rater. Subsequently, the researchers conducted verbatim coding of the research themes, the impact factor of ethics education, and the main research populations based on the content reported in the research articles. After establishing the coding scheme for the systematic review of the 145 selected articles, the two raters coded the remaining literature separately and compared their coding results. After the independent coding (the Kappa statistics were 0.88), a satisfactory agreement was achieved with differences resolved via discussion. No ethical approval is required for this review.

## 3 Results

**FIGURE 1:**
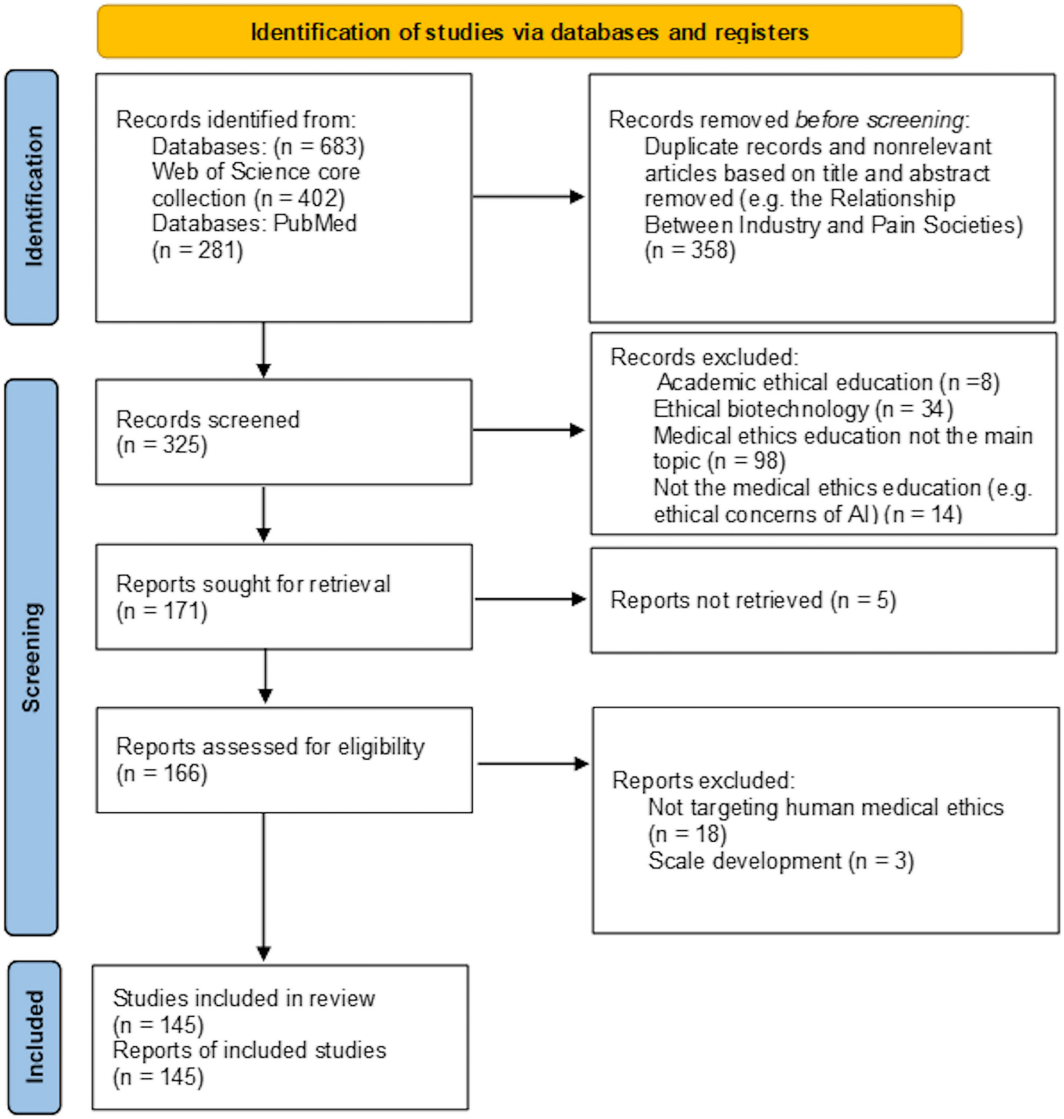
PRISMA flow diagram.

A total of 683 records were identified in database searches after using date and language limits, 145 articles to be assessed as full texts in the final. Our coding exercise generated four main themes and seven sub themes of the moral education for medical learning or ethics education for medical learning. The Preferred Reporting Items for Systematic Reviews and Meta-Analyses (PRISMA) flow chart shows the details of the search process (see Figure 1).

### 3.1 Synthesis results

This study divided the full text of 145 publications into four themes, including virtues-based ethics education, competency-based ethics education, professionalism, and mutual safety among medical students and healthcare professionals. 102 publications solely discussed one themes, and 44 publications mentioned two or above themes (the details see Table 2). Among these, competency-based ethics education was the area most frequently discussed in the literature (*N* = 58, 40%), while virtues-based ethics education and professionalism are the most closely related (*N* = 18, 12.41%).

**TABLE 2:** Descriptive results.

Theme	N
Virtues-based ethics education	12
Competency-based ethics education	58
Professionalism	28
Mutual safety	4
Virtues-based ethics education and competency-based ethics education	5
Virtues-based ethics education and professionalism	18
Virtues-based ethics education and mutual safety	1
Competency-based ethics education and professionalism	13
Competency-based ethics education and mutual safety	3
Professionalism and mutual safety	1
Virtues-based ethics education, competency-based ethics education and professionalism	2
Total	145

### 3.2 Competency-based ethics education

The competency-based medical education (CBME) approach has gained much attention in healthcare professions’ education in the recent 10 years. This trend also presents in the medical ethical fields. 55.86% (81/145) publications discussed competency-based ethics education (Table 3). Aligning to the CBME model, research indicated that aims to enhance health care by emphasizing longitudinal learning and the key competencies of clinical ethics can be trained by education and practice (19). To determine the inner dimensions of competency-based ethics education, James Rest’s Four-Component Model was adopted to analysis. It conceptualizes moral behavior as a sequential psychological process comprising moral sensitivity (recognizing ethical issues), moral judgment (evaluating right and wrong), moral motivation (prioritizing moral values), and moral action (executing behavior), asserting that all components must interact concertedly for ethically complete conduct (20). Shed light on this model, it can help us understand how to develop the behavior, competence, and skills of medical students in different mental processes.

**TABLE 3:** Summary of competency-based ethics education.

Sub themes	Inner dimensions	Key features	References
Abilities (*N* = 40)	The ability of moral sensitivity (*N* = 14)	Recognize the related clinical ethical issues; acknowledge and accept uncertainty; the specific clinical ethics of surgical ethics; ethics education; knowledge in clinical ethics; behavior, competence, and skillss	Coskun et al. (37); Allana et al. (38); Olaiya et al. (39); Subedi et al. (40); Liu et al. (41); Nadolny et al. (42); Wan et al. (8); Tonelli and Upshur (21); Liu et al. (43); Amendola et al. (44); Lin et al. (45); Althagafi and Alahmad (46); Shamim et al. (47); Hertrampf et al. (48)
	Critical thinking and ethical reasoning abilities (*N* = 14)	Critical thinking, moral reasoning, moral choices, clinical reasoning and moral judgment, moral courage, the ability to analyze ethical situations, professional ethical decision making	Kalet et al. (49); Amar-Gavrilman and Bentwich (50); Jean-Tron et al. (51); McDonald et al. (52); Mahdavifard et al. (53); Kaldjian et al. (54); Ribeiro et al. (55); Savitha et al. (56); Barman et al. (57); Kelly et al. (58); Sanatani and Muir (59); Gangwani et al. (60); Landa-Galindez et al. (61); McLean and Bottrell (62)
	Empathy (*N* = 12)	Compassion, empathetic communication; empathic care; empathic response; empathy erosion; empathy-burnout relationship; cognitive empathy	Cheu et al. (63); Chao et al. (22); Assing Hvidt et al. (23); Prabhath et al. (64); Rawal et al. (65); Pieris et al. (66); Hizomi Arani et al. (67); Pal et al. (19); Dodsworth et al. (24); Ortiz-Paredes et al. (9); Anto and Savitha (68); Benbassat (69)
Behaviors (*N* = 25)	Moral behavior (*N* = 3)	Reactionary behavior, prosocial behavior, moral distress	Paros and Tilburt (34); Jin et al. (33); Perni et al. (70)
	Insufficient communication (*N* = 4)	Informed consent, communication	Pai et al. (71); Friesen et al. (28); Kaur et al. (29); Zuchelkowski et al. (30)
	Bias and discrimination (*N* = 11)	Bias, healthcare for the stigmatized patients, healthcare for the vulnerable patients,	de Vries et al. (25); Nemli et al. (72); Hashmi et al. (73); Sukhera and Watling (74); Liu et al. (75); To et al. (76); Lowik et al. (77); Wellbery et al. (26); Arazi et al. (27); Jegan and Dierickx (12); Camp et al. (78)
	Systems-based practice (*N* = 7)	Structural moral distress, hidden curriculum and institutional norms, laws and regulations, cross-cultural training; international guidelines	Wijma et al. (79); Greenberg et al. (32); Fan et al. (80); Shiao et al. (31); Sukhera et al. (81); Bleicher et al. (82); Tekian et al. (83)
Skills (*N* = 16)	Communication skills (*N* = 9)	Communication skills, mediation skills with patients, communication attitude, patient management skills; interpersonal skills	Nayak et al. (35); Yaylaci et al. (84); Bin Abdulrahman et al. (85); Antonsen et al. (86); Chen et al. (87); Aoun et al. (88); Torda (89); Williams et al. (90); Dickinson et al. (91)
	Ethical skills (*N* = 7)	Humanities-based skills, moral reasoning skills, ethical skills	Delbani et al. (92); Leffel et al. (93); Magalhães-Sant’Ana (17); Hewko et al. (36); Marei et al. (94); Dong et al. (95); Al Suwayri (96)

**FIGURE 2:**
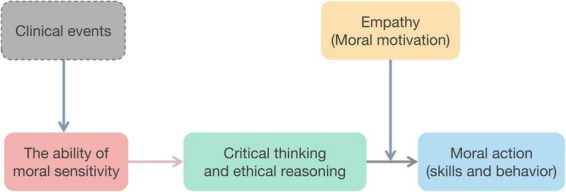
The framework of inner dimensions of competency-based ethics education.

The Four-Component Model conceptualizes morally complete behavior as a sequential process, including moral sensitivity, moral judgment, moral motivation, and moral action. This study adopted on this framework, and developed the cultivation of medical behavior, abilities, and skills as a set of psychological processes. In the first step of the ability of moral sensitivity, educators must train medical students to recognize clinical ethical issues and acknowledge uncertainty in ethics education to improve their moral sensitivity (21). Then, in the second step of critical thinking and ethical reasoning abilities, cultivating deliberation on ethically sound choices and consequences can help medical students enhance their critical thinking and make moral judgment in clinical settings. Thirdly, empathy is crucial for moral motivation. The declined empathy can negatively impact moral judgment and bring bias and discrimination in the subsequent behavior. In the final step, medical students’ moral action can be actualized through deliberate skills training (e.g., ethical decision-making, communication) and reinforced moral behavior (e.g., prosocial conduct, bias mitigation), modulated by systems-based practices that enable or constrain ethical agency (Figure 2).

#### 3.2.1 Sub themes of abilities

27.59% (40/145) publications described the abilities in ethical education, which accounts for nearly half of competency-based ethical education (40/81, 49.38%). There are three inner dimensions in previous studies, including the ability of moral sensitivity, critical thinking and ethical reasoning abilities, and empathy. Based on James Rest’s Four-Component Model (20), the ability of moral sensitivity is the first step of moral behavior. This kind of moral sensitivity can help health professions raise their awareness of self-care to relief moral distress (8) and deal with the clinical dilemmas. Moral judgment is the second process of the Four-Component Model, it emphasizes deliberating on ethically sound choices and their potential consequences. Recent studies suggested that empathy can be viewed as a learnable and modified psychological construct through educational interventions (9, 19), which can motive health professions’ subsequent behavior. However, researchers found that empathetic communication (22) and empathic care (23) are lacking in the clinical curriculum. A decline in empathy has a relationship with burnout and ethical erosion (24).

#### 3.2.2 Sub themes of behaviors

There were four inner dimensions of behavior, including bias and discrimination, systems-based practice, insufficient communication, and moral behavior. The most concern behavior was bias and discrimination, which significantly affects patient care and the professional ethics (25). The stereotypes were the main reasons to bias and discrimination for healthcare professions (26, 27). Four studies focus on the problem of informed consent and communication between doctors and patients (28–30). Beyond the individuals’ perspective, cultural background also impacted on moral behavior in clinical setting (31, 32). Therefore, systems-based practice profoundly shapes medical ethics by perpetuating inequities, silencing dissent, and constraining moral agency. To reduce this phenomenon, Jin et al. (33) found that promoting prosocial behavior through medical curricula can reduce moral distress and increase the positive effect. Additionally, Paros and Tilburt (34) indicated that the integration of the moral intuitionist model into medical ethics education can improve medical students respond to behavior they may find immoral or misguided.

#### 3.2.3 Sub themes of skills

Skills were the least mentioned sub themes of competency-based ethics education (19.75%, 16/81). On one end, communication skills were emphasized for healthcare professions’ training [e.g., (35)]. On the other end, educators and aimed to improve healthcare professions’ ethical skills to help their clinical work. To be noticed, there are something difference between moral reasoning ability and moral reasoning skills. The former mainly focuses on developing medical students’ critical thinking in ethical dilemmas within ethical deliberation processes, whereas the latter is associated with the skill set to make ethical decisions effectively (36).

### 3.3 Virtues-based ethics education

Unlike competency-based ethics education, virtues-based ethics education is the traditional theme of medical ethics education. 26.21% (38/145) publications discussed virtues-based ethics education (Table 4). Several researchers suggested that certain affective components of medical attributes may be innated (19), which is conceptualized ethical virtues (97) and characters (98). Virtue is viewed as the moral qualities or tendencies of an individual that promote moral behavior, including humanistic care and virtues ethics such as integrity, respect, and trust (54, 99). Characters, on the other hand, focus more on an individual’s moral construct (100, 101).

**TABLE 4:** Summary of virtues-based ethics education.

Sub themes	Inner dimensions	Key features	References
Virtues (*N* = 31)	Moral wisdom (*N* = 10)	Practical wisdom, critical thinking, acting wisely, moral courage et al.	Ribeiro et al. (107); Chang et al. (108); Hughes and Rushton (109); Kaldjian et al. (54); Verstegen et al. (103); Hawking et al. (102); Beck et al. (110); Muhaimin et al. (3); Hlaing et al. (111); Dornan et al. (18)
	Virtue development (*N* = 8)	Self-Reflection, moral resilience; dignity, justice, equity, internalization of ethical principles; humanism et al.	Ghodousi et al. (112); Bäckryd (99); Magalhães-Sant’Ana (17); Dowie (97); Vilagra et al. (113); Dingle and Kolli (114); Kim et al. (104); Okamoto et al. (115)
	Humanistic care and virtues ethics (*N* = 8)	Beneficence, humanism, compassion, integrity, humility; ultimate concern; fairness, justice et al.	McDaniel et al. (116); Guo et al. (117); Stephens et al. (118); Liao (119); Goss et al. (120); D’Angelo et al. (121); Karunakaran et al. (122); Leffel et al. (93)
	Professional virtues (*N* = 5)	Confidentiality, obligation to the profession, patient welfare, respect, autonomy, honesty, trust, et al.	Greiner and Kaldjian (123); Mogodi et al. (101); AlMahmoud et al. (124); Jegan and Dierickx (12); Allana et al. (38)
Character (*N* = 7)	The character of “Good doctor” (*N* = 3)	Positive psychology’s framework of character strengths, professionalism, moral intuition to care et al.	Cianciolo et al. (5); Aoun et al. (88); Leffel et al. (105)
	Moral character and personality traits (*N* = 4)	Altruism, self-esteem, optimism, control, self-discipline, service and sacrifice et al.	Haddara and Lingard (100); Hur et al. (98); Pal et al. (19); MacKenzie et al. (106)

**FIGURE 3:**
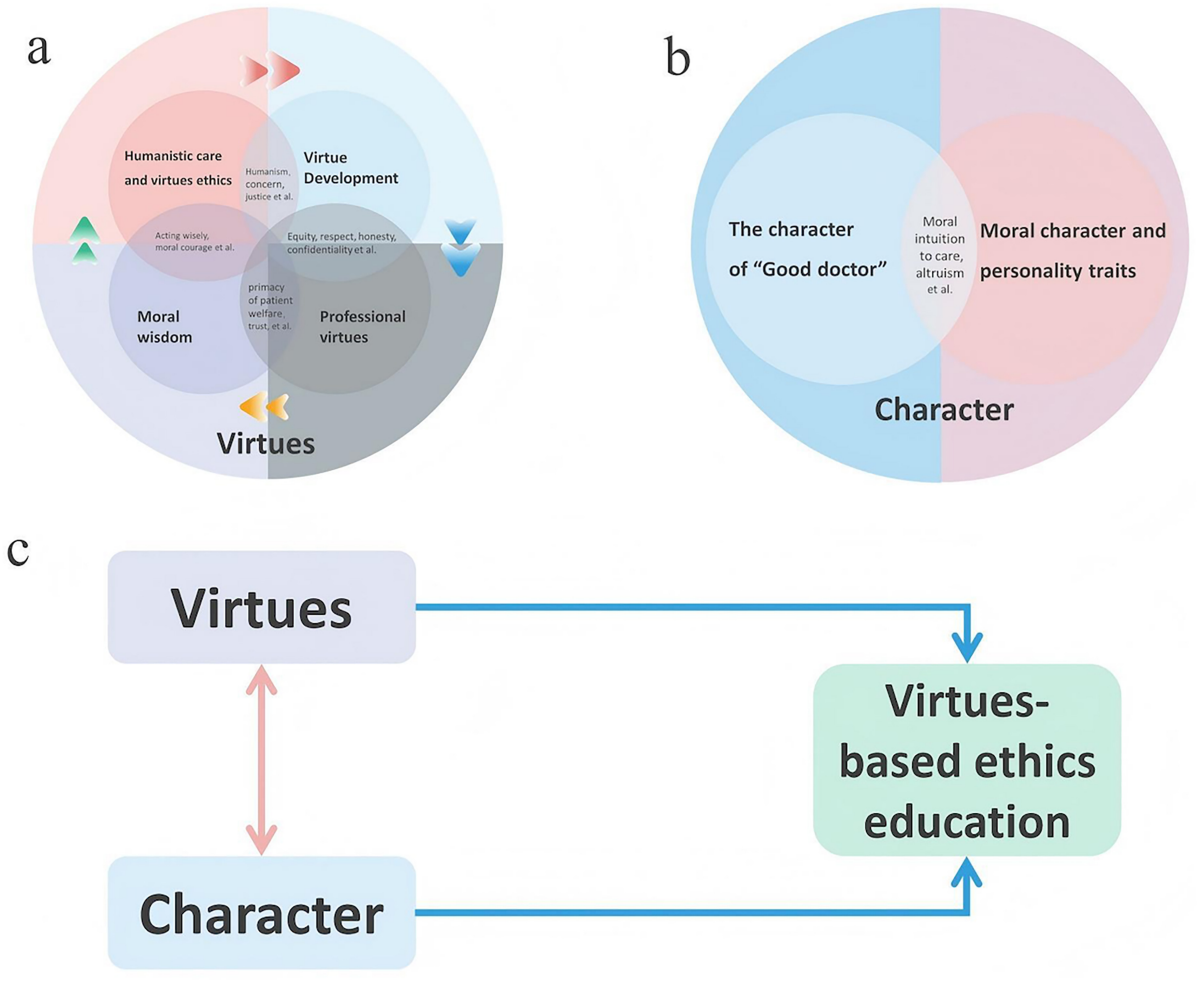
The framework of inner dimensions of virtues and characters. **(a)** The four inner dimensions of virtues; **(b)** the two inner dimensions of character; **(c)** the relationship between virtues and character.

Medical education strives to cultivate physicians with humanistic virtues, which manifests as concrete professional virtues. Physicians must possess moral wisdom to implement these virtues in complex real-world scenarios. Ultimately, the establishment and deepening of all such virtues depend on virtue development permeating the entire educational process. It is worth noting that there is not a linear relationship, but an interrelated relationship among the inner dimensions of virtue. Moral character and personality trait are the cornerstone of a “Good Doctor” (e.g., moral intuition to care). Both humanistic virtues and professional virtues are the ethical substance to shape the character of “Good doctor.” Each inner dimension (e.g., from professional virtues to good doctor character) forms a feedback loop that can continuously promote the virtues development of clinicians to acting wisely in the complex clinical environments (Figure 3).

#### 3.3.1 Sub themes of virtues

Previous studies on medical ethics education conceptualize the theme of virtues through four dimensions: moral wisdom, virtue development, humanistic care and virtue ethics, and professional virtues (Table 4). Moral wisdom is the most prominently discussed dimension (32.26%, 10/31), defined as a specialized form of practical wisdom essential to deal with the complex moral clinical environments (54). Cultivating this wisdom enables health professionals to act judiciously (18) and discover optimal approaches to achieve patients’ wellbeing (54, 102, 103). Closely related is the ongoing process of virtue development (29.03%, 9/31), which involves internalizing the moral qualities of healthcare professionals (17). Scholars emphasize that medical learners must engage in self-reflection to evolve as moral agents striving for clinical excellence (104).

#### 3.3.2 Sub themes of characters

Among all the sub-themes, character received the least attention. Only 4.83% of publications (7/145) discussed the character education of healthcare professionals. In the sub themes of character, there are two inner dimensions of character in pervious study of medical ethics education, which is the character of “Good doctor” and moral character and personality traits. On the other hand, virtues provide an organized, meaningful structure for understanding the diverse array of character strengths. Virtues are broad, universally recognized categories of moral excellence, while character strengths are the specific psychological traits. When medical education published the Project on the Good Physician, researchers aimed to find out what character should the “Good doctor” to be. However, only three publications mentioned the sub themes (2.07%, 3/145). Researchers proposed the moral intuitionist model (105) and the positive psychology’s framework to enhance medical professionals’ engagement and wellbeing (5). Positive psychology may offer a framework for researchers to understand the relationship between virtues and character. On one hand, researchers claim that “constellation of characteristics manifest in virtuous dispositions and practices” (5). These studies emphasize that character education is particularly important for medical doctors compared with other professions (19, 98, 100, 106).

### 3.4 Professionalism

**FIGURE 4:**
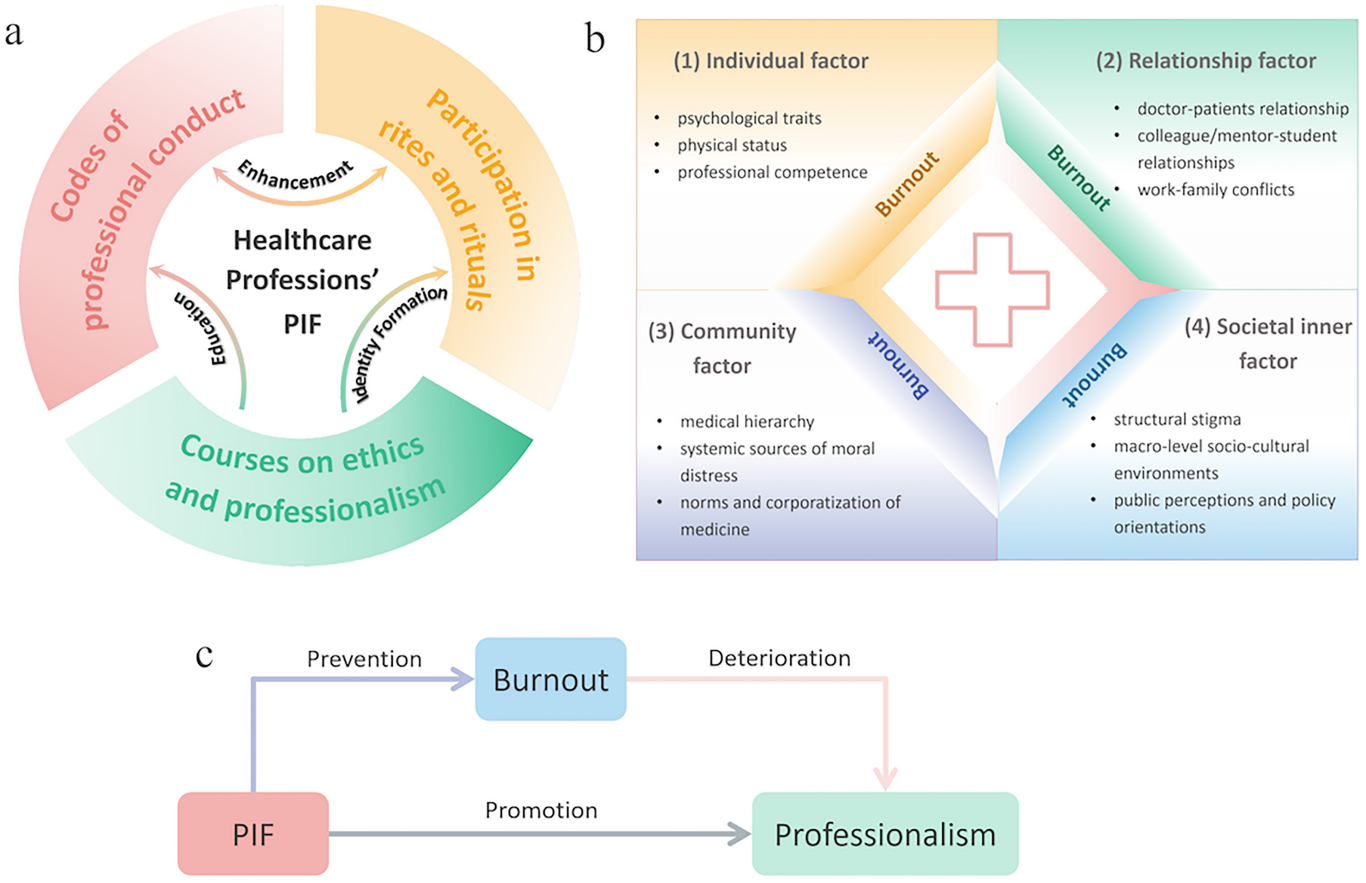
The framework of inner dimensions of professionalism. **(a)** The three inner dimensions of PIF; **(b)** the four inner dimensions of burnout; **(c)** the relationship between PIF and burnout.

The data indicated that virtues-based ethical education has a close relationship with professionalism. Professionalism was the second most common theme in healthcare professions’ ethics education. 62 out of 145 publications mentioned professionalism (42.76%). Professional identity formation (PIF) and burnout were the two sub-themes in professionalism (Table 5). Professional identity formation (PIF) is a continuous, integrative process shaped by courses, experiences, socialization, and reflection (113, 125). Cruess et al. (126) proposed that when doctors internalize their professional values, work pressure will be transformed into a sense of mission rather than a burden. Conversely, the burnout experienced will negatively affect their professionalism. If PIF can be well formed, burnout may be prevented in subsequent clinical work. It should not be ignored in healthcare professionals’ clinical work. Researchers found that vocational calling and identity were significantly negatively correlated with burnout (105) (Figure 4).

**TABLE 5:** Summary of professionalism.

Sub themes	Inner dimensions	Key features	References
PIF (*N* = 50)	Courses on ethics and professionalism (*N* = 22)	The professionalism curriculum, ethics and professionalism teaching, professional identity and identity integration, professional identity development, professionalism instruction and assessment, professional capabilities, professional duties, the traditional concept of In-Sul	Hawking et al. (102); Martimianakis et al. (7); Bin Abdulrahman et al. (85); Allana et al. (38); AlMahmoud et al. (124); Park et al. (133); Buck et al. (134); Liu et al. (41); Schei et al. (135); Hlaing et al. (111); Burla et al. (136); Kaldjian et al. (54); Reimer et al. (137); Ribeiro et al. (107); Irby and Hamstra (131); Barman et al. (57); Verstegen et al. (103); Dilday et al. (138); DiBrito et al. (139); McLean and Bottrell (62); Gangwani et al. (60); Muhaimin et al. (3)
	Codes of professional conduct (*N* = 23)	Critical thinking, moral reasoning, moral choices, clinical reasoning and moral judgment, moral courage, the ability to analyze ethical situations	Kalet et al. (49); Liang et al. (6); Keshavarzi et al. (140); Alkahtani et al. (141); Fu et al. (142); Bashir et al. (125); Vilagra et al. (113); Ribeiro et al. (55); Wald and Ruddy (130); Sebastian et al. (128); Woolley et al. (143); Dingle and Kolli (114); Kaur et al. (29); Colbert-Getz et al. (144); Chen et al. (129); Grundnig et al. (145); Links et al. (146); Helmich et al. (147); Brown et al. (148); Emmerich (149); McLean et al. (150); Bäckryd (99); Kim et al. (104)
	Participation in rites and rituals (*N* = 5)	Medical oaths, memorial ceremony in medical anatomy courses	Goss et al. (120); Greiner and Kaldjian (123); Karunakaran et al. (122); Stephens et al. (118); Madgwick et al. (151)
Burnout (*N* = 12)	Individual factor (*N* = 5)	Positive psychology’s framework of character strengths, prosocial behavior, empathy, non-technical attributes, moral intuition to care of medical character	Cianciolo et al. (5); Jin et al. (33); Ortiz-Paredes et al. (9); Lin et al. (45); Leffel et al. (105)
	Relationship factor (*N* = 3)	Healthcare for the special populations, ineffective communication with patients	Perni et al. (70); Antonsen et al. (86); Nishigori et al. (152)
	Community factor (*N* = 2)	Medical hierarchy, systemic sources of moral distress, norms and corporatization of medicine, hierarchical system of decision-making	Humphrey et al. (153); Beck et al. (110)
	Societal inner factor (*N* = 2)	Structural stigma, pandemic	Sukhera et al. (81); Hughes and Rushton (109)

#### 3.4.1 Sub themes of PIF

34.48% (50/145) publications discussed PIF, which was the most frequently mentioned sub themes of all. In the PIF, researchers proposed three inner dimensions to promote PIF, including courses on ethics and professionalism, codes of professional conduct, and participation in rites and rituals (127). Researchers’ attentions were mainly captured by the two dimensions, respectively courses on ethics and professionalism and codes of professional conduct. In the course perspective, researchers aimed to set the formal and hidden curriculum of professionalism curriculum (7, 16) to boost PIF during their studies. In the perspective of professional conduct, researchers aimed to set professional conduct to shape healthcare professions’ behavior to enhance their professionalism (125, 128–130). For example, positive mentoring, effective feedback, and longitudinal coaching offer medical students or trainees’ professional conduct as they progress toward becoming a “good doctor” (127, 131).

#### 3.4.2 Sub themes of burnout

Burnout can negatively impact on professionalism of medical students or medical professionals. In the burnout, based on the Socio-ecological Model for Burnout Prevention for health workers (132), including individual, relationship, organizational, community, and societal inner dimensions, that can influence burnout. Individual factor in healthcare professions’ burnout was mentioned most frequently (41.67%, 5/12), while higher-level factors (e.g., community factor and societal inner factor) were mentioned less frequently.

### 3.5 Mutual safety

6.21% (9/145) publications discussed mutual safety in medical ethics education, which is the least mentioned theme. Researchers recently outline the concept of “mutual safety,” increasing patients’ benefit (154) as well as reduce harm in complex clinical situations. The healthcare professions also can benefit from mutual safety and keep a balance in doctor-patients relationship (18). While educating doctors to abide by moral principles and scientific technologies established medicine as a profession, applying simplistic standards to an increasingly complex practice can make them feel more stressed. It may explain healthcare professions’ deteriorating mental health. There is a new threat for medical trainees and healthcare workers (155). Therefore, it is a growing key theme to introduce “mutual safety” into medical ethics education to promote restoration of the doctor-patient relationship (156). Mutual safety may relate with the traditional Eastern value of In-Sul (benevolent art) in the medical culture (133), which has long emphasized reciprocal ethical obligations in human relationships. Clinicians gained confidence, intrinsic motivation, satisfaction, capability, and a sense of legitimacy from acting wisely of benefiting patients, which ensures the healthcare professions’ psychosocial safety and patient safety (18). Mutual safety is essential for effective patient care and conceptualizing medical professionalism (21, 64, 133, 157), and empathy is important to promote this relationship (65).

### 3.6 Integrating multidimensions of four themes

**FIGURE 5:**
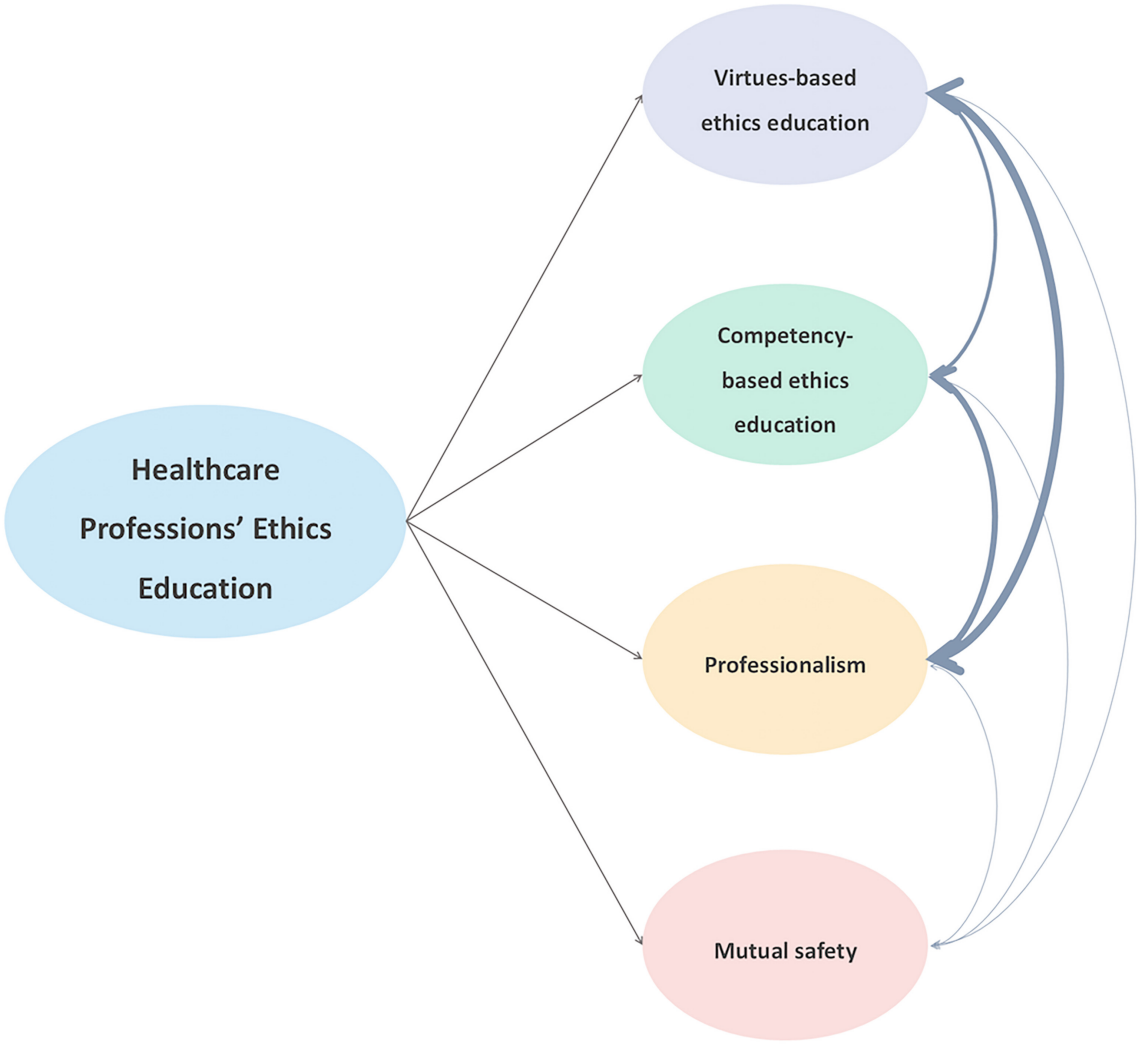
The relationship of four themes. The thickness of the mutual arrow lines connecting the themes represents the closeness of the relationship and the frequency of interaction between them.

This study indicated that 43 publications mentioned two or above themes (the detailed data in Table 2). The data showed that virtues-based ethics education and professionalism is the most active interrelationship (Figure 5). Professional identity forms when professional virtues are internalized through clinical experiences. Professionalism emerges from aspirations to the character of a “good physician” rather than merely conforming to norms (5). There were 2 publications simultaneously discussed three themes, exploring the interrelationships among virtues-based ethics education, competency-based ethics education, and professionalism. During the clerkship, moral decisions in dilemmas were important experiences that can impact on the identity formation of trainee and cultivate in development in moral courage (55). For example, ineffective communication with patients can bring distress, low job satisfaction, and burnout to healthcare professions (86). Additionally, system-level pressure of norms and corporatization of medicine and personal burnout can erode their virtues and mislead moral behavior and bring a barrier to form healthcare professions’ identity integration (110). The importance of mutual safety has been proposed in recent years (18). It not only educates doctors to abide by moral principles or to be a good doctor but also lies in addressing complexity called for a skills-set to shape a good doctor-patients relationship (155–157).

## 4 Discussion

In the current systematic review study, we examined the way in which the themes of medical ethics education have been understood and integrated into the ethics training of health professions. To best our knowledge, no previous publications have systematically explored into these integrating aims of human medical ethics education. In addition, the current study included the perspective of medical students, medical teachers and patient teachers, which allows us to approach medical ethics education from a more comprehensive perspective. By involving a diverse group of participants, we can gather a range of insights and experiences that reflect the multifaceted nature of ethical education in healthcare. Therefore, the current systematic review makes a significant contribution to the available literatures in the framework of medical ethics education.

### 4.1 The emerging trends of healthcare professions’ ethics education

First, as the major finding, regarding the theme of medical ethics education, the data indicated that the researchers were mostly concerned with competency-based ethics education. There is a growing interest in competency-based medical education models (19). Like medical education, the current medical ethics education is also based upon a competency-centered framework (53). Ethics education emphasizes the competency of health professions’ non-technical attributes (45), enabling them to effectively apply acquired knowledge and skills in practical healthcare settings. Specifically, ethical competency development focuses on specific skills, such as patient communication, clinical judgment, and ethical decision-making. All of these are essential competence that medical students must master during clinical practice (84). Researchers found that medical students’ ethical development can be enhanced through applying their moral competency in complex clinical ethical scenarios (111). Furthermore, researchers suggested that students’ moral development is better enhanced by the cognitive approach of skills-based ethics programs, as opposed to purely emotional approaches. This explains why competency-based ethics education has been so popular in literature over the last decade. In contrast, while the cultivation of virtue is important (102), its abstract nature and emphasis on intrinsic values often make it difficult to translate into quantifiable outcomes in the short term.

As a second major finding, PIF emerged as the most frequently mentioned sub themes among the reviewed publications. The current study explores the dynamic interplay between PIF, burnout, and professionalism. In this context, medical professionalism is conceptualized as a representation of the self, achieved in stages over time as the characteristics, values, and norms of the medical profession are internalized (49). The first path is that PIF can directly enhance professionalism, while the second path is that burnout can negatively affect it (5). However, previous researchers have placed less focus on how burnout negatively affects professionalism. By employing the Socio-Ecological Model to analyze healthcare worker burnout (132), we found that individual, relational, community, and societal inner factor can contribute to varying degrees of burnout. This burnout is frequently associated with clinical practice during clerkships or later career stages (70, 81), diminishing physician empathy (9). Because developmental theorists emphasize that identity formation is a continuous, lifelong process (49), it is important for educators and researchers to prioritize strategies that mitigate burnout and promote work-life balance in clinical settings (152).

As a third major finding, this review highlighted the concept of mutual safety in medical ethics education. Mutual safety is one of the recently introduced education goals in medical ethics education. As it is recently outlined, many educators and researchers may not have fully integrated their content into existing educational frameworks. However, mutual safety plays an important role in the complexity of practice healthcare professions and patients’ safety (18). It provided researchers with a new perspective to investigate how healthcare professions can benefit from reducing patients’ harm. It can shape a new model of doctor-patient relationship (64), which can also enhance empathetic communication (22). Recently, an earlier publication supported the concept of mutual safety that intolerance of uncertainty brings to uncertainty, ambiguity, and patients’ overtreatment, reducing the benefit of physicians (21). Applying the concept of mutual safety in medical ethics education, researchers argue the multidimensional virtue of practical wisdom (54, 102) can help health professions benefit patients amidst complexity (18).

Notably, as a fourth major finding, the current study outlines the dynamic relationships among the four core themes of healthcare ethics education. While previous evidence indicates an overlap among these themes (38, 54), there remains a lack of a 10-year systematic review examining their interrelationships. This study provides a more comprehensive framework of medical ethics to achieve the education goal. We proposed that medical students and health professionals should learn and practice by applying the concept of mutual safety to form their professionalism. Competency-based ethics education answers the question of “what a physician should be able to do,” professionalism answer the question of “how to be a professional physician” (e.g., codes of professional conduct). Then, what framework answer the question of “who a physician should be”? This answer may be underly in virtues-based ethics education and professionalism. Although competency-based ethics education has been extensively investigated, the virtues approach is still the traditional teaching goal in medical ethics. A primary goal of medical education is to facilitate professional identity formation, shaping students and professionals to think, feel, and act in accordance with health professions (136). The data also supported that the interaction frequency between virtues-based ethics education and professionalism is the most active. This interaction not only emphasized in formal courses (104), but also in hidden courses (122). For example, gross anatomy courses are crucial for fostering self-reflection among medical students on their roles as healthcare professionals, highlighting that the hidden curriculum of tacit learning significantly shapes their training in humanistic care and virtue ethics (7, 117). This process relies on teaching knowledge, codes of professional conduct, practicing beliefs, and their virtues and character.

### 4.2 Future study and limitations

In the future, research on medical ethics education should aim to build a comprehensive framework that integrates virtues-based ethics education, competency-based ethics education, professionalism, and mutual safety, focusing on exploring the dynamic interaction mechanisms among these themes. In the future study, empirical studies or longitudinal studies are required to examine the causal relationship of four themes. Lastly, against the backdrop of rising digital and AI-based healthcare, it is also essential to consider how to apply traditional ethical principles to the moral challenges posed by emerging technologies (115).

There are some limitations in this PRISMA study. The first concern is that the study only searches the publications from Web of Science. This results in the database retrieved not being comprehensive. However, we believe that the retrieved publications are the high-quality publications in medical ethics education. The second limitation related to the way in which medical ethics are closely associated with local culture and law, which gives lesser consideration in this review. Lastly, focus upon guidelines published in English might potentially exclude some high-quality studies published in other languages.

## 5 Conclusion

Medical ethics education plays an important role in clinical practice and moral decision-making. This review integrated four themes of the emerging trends in healthcare professions’ ethics education, which will be helpful for AI education in medical ethics education. In this study, it is the first time to provide a more comprehensive framework of medical ethics to achieve the education goal. The data indicated that the dynamic relationship among these four themes suggests a strong and close connection among them. Additionally, mutual safety is one of the recently introduced education goals in medical ethics education. Researchers should conduct empirical studies or longitudinal studies to explore it.

## Data Availability

The raw data supporting the conclusions of this article will be made available by the authors, without undue reservation.
