# Supplementary material for: The emerging trends of healthcare professions’ ethics education in recent 10 years: a systematic review
Source: Front Med (Lausanne). 2026 Mar 17;13:1748761. doi: 10.3389/fmed.2026.1748761 (PMC13037488; doi:10.3389/fmed.2026.1748761)
Supplement: Supplementary file 1 [file Data_Sheet_1.pdf]

Supplementary Material

Table S1 Search Strategies for Web of Science and Pubmed databases

Database	Search Strategy	Number of Results	Filers	Date
Web of Science	The following search strategy adopted a combination of two topics (topic 1: moral education or ethics education; topic 2: medical). The terms were combined using Boolean statements (moral education or ethics education and medical). Key categories included education scientific disciplines, educational research, psychology in education, and special education.	402	Science Citation Index (SCI) and Social Sciences Citation Index (SSCI)<br><br>English<br><br>2015-2025	April 1st, 2025
Pubmed	The specific search strategy (Query box of Advanced Search Builder) executed in PubMed is detailed below: (("Morals"[Mesh] OR "Ethics"[Mesh] OR "Bioethical Issues"[Mesh] OR "moral education"[tiab] OR "ethics education"[tiab] OR "ethical reasoning"[tiab]) AND ("Education"[Mesh] OR "Education, Medical"[Mesh] OR "Curriculum"[Mesh] OR teaching[tiab] OR learning[tiab]) AND ("Education, Medical"[Mesh] OR "Physicians" [Mesh] OR "Students, Medical"[Mesh] OR medical[tiab] OR medic*[tiab] OR physician*[tiab] OR doctor*[tiab])).	281	Science Citation Index (SCI) and Social Sciences Citation Index (SSCI)<br><br>English<br><br>Last 10 years	April 1st, 2025

Table S2 Example of coding table

Research questions: (1) what are the emerging trends of healthcare professions' ethics education in recent 10 years?
(2) what is the interrelationship between the various goals of the emerging trends of healthcare professions' ethics education?

Num.	Publications	Virtues-based Ethics Education						competency-based ethics education											Professionalism							Mutual safety
		Virtues				Character		Abilities			Behaviors				Skills		PIF			Burnout				
		Moral wisdom	Virtue development	Humanistic care and virtues ethics	Professional virtues	The character of “Good doctor”	Moral character	The ability of moral sensitivity	Critical thinking and ethical reasoning abilities	Empathy	Moral behavior	Insufficient communication	Bias and discrimination	Systems-based practice	Communication skills	Ethical skills	Courses on ethics and professionalism	Codes of professional conduct	Participation in rites and rituals	Individual factor	Relationship factor	Community factor	Societal inner factor	
1	Massé et al., (2024)	0	0	0	0	0	0	0	0	0	0	0	0	0	0	0	0	0	0	0	0	0	0	1
2	Ribeiro et al. (2025)	1	0	0	0	0	0	0	0	0	0	0	0	0	0	0	1	0	0	0	0	0	0	0
3	Dowie (2023)	0	1	0	0	0	0	0	0	0	0	0	0	0	0	0	0	0	0	0	0	0	0	0
4	Sukhera et al. (2018)	0	0	0	0	0	0	0	0	0	0	0	1	0	0	0	0	0	0	0	0	0	0	0
5	Wald et al., (2021)	0	0	0	0	0	0	0	0	0	0	0	0	0	0	0	0	1	0	0	0	0	0	0
6	Liu et al., (2022)	0	0	0	0	0	0	0	0	0	0	0	1	0	0	0	0	0	0	0	0	0	0	0
7	Hertrampf et al., (2019)	0	0	0	0	0	0	1	0	0	0	0	0	0	0	0	0	0	0	0	0	0	0	0
8	Verstegen et al., (2023)	1	0	0	0	0	0	0	0	0	0	0	0	0	0	0	1	0	0	0	0	0	0	0

Notice: 8 studies as examples for coding. As illustrated in the table, we employed a clear binary coding system to map the literature: “1” indicates the mention of a sub-theme within a publication, while a “0” indicates its absence.

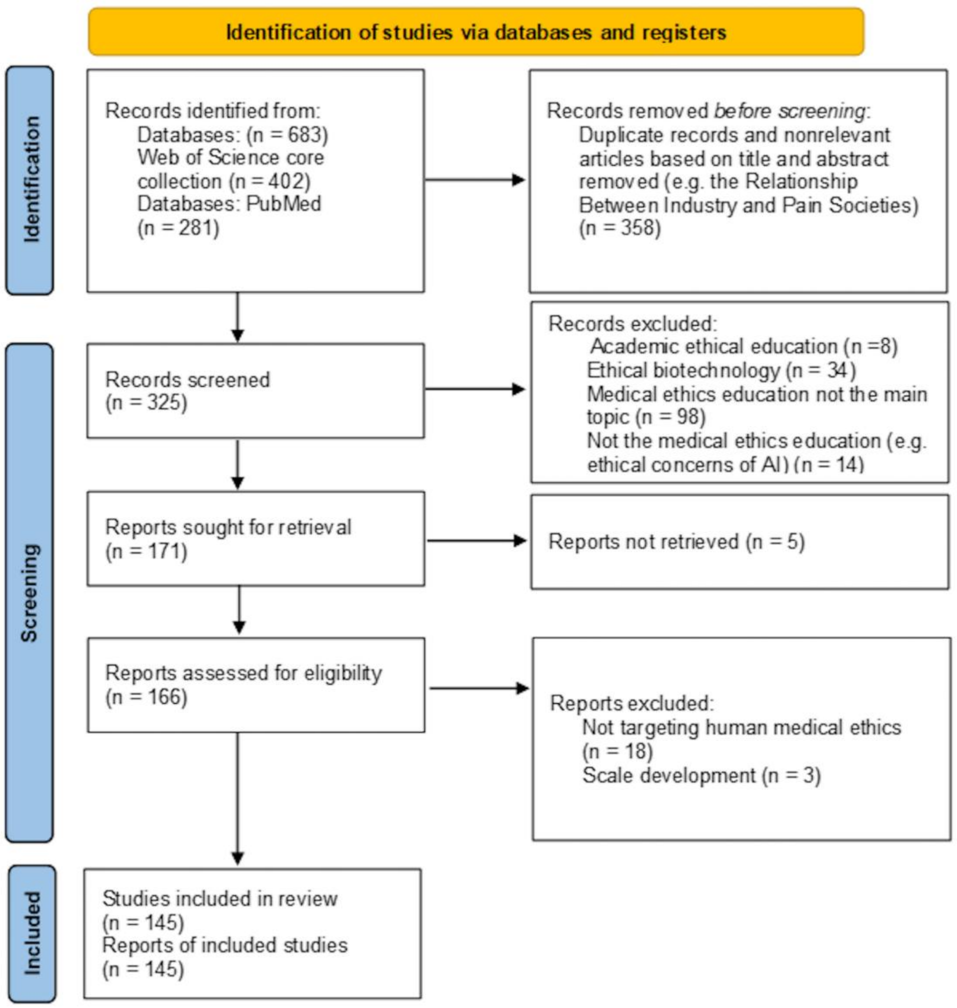

Figure 1 PRISMA flow diagram

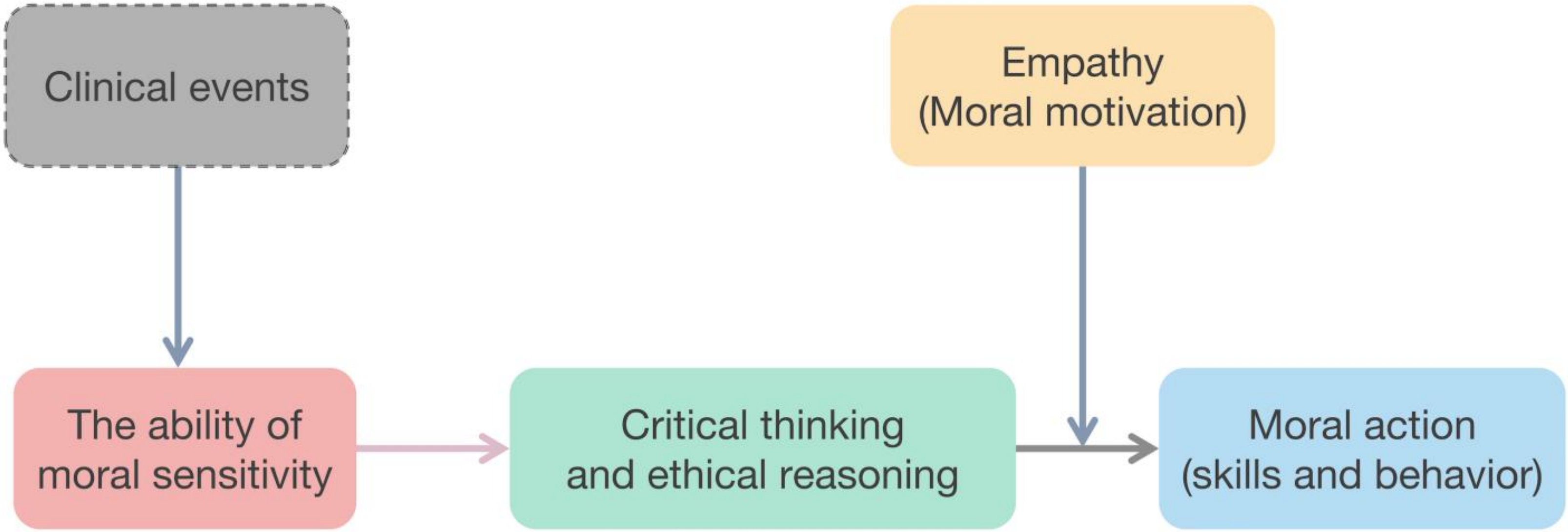

Figure 2 The framework of Inner Dimensions of Competency-based Ethics Education

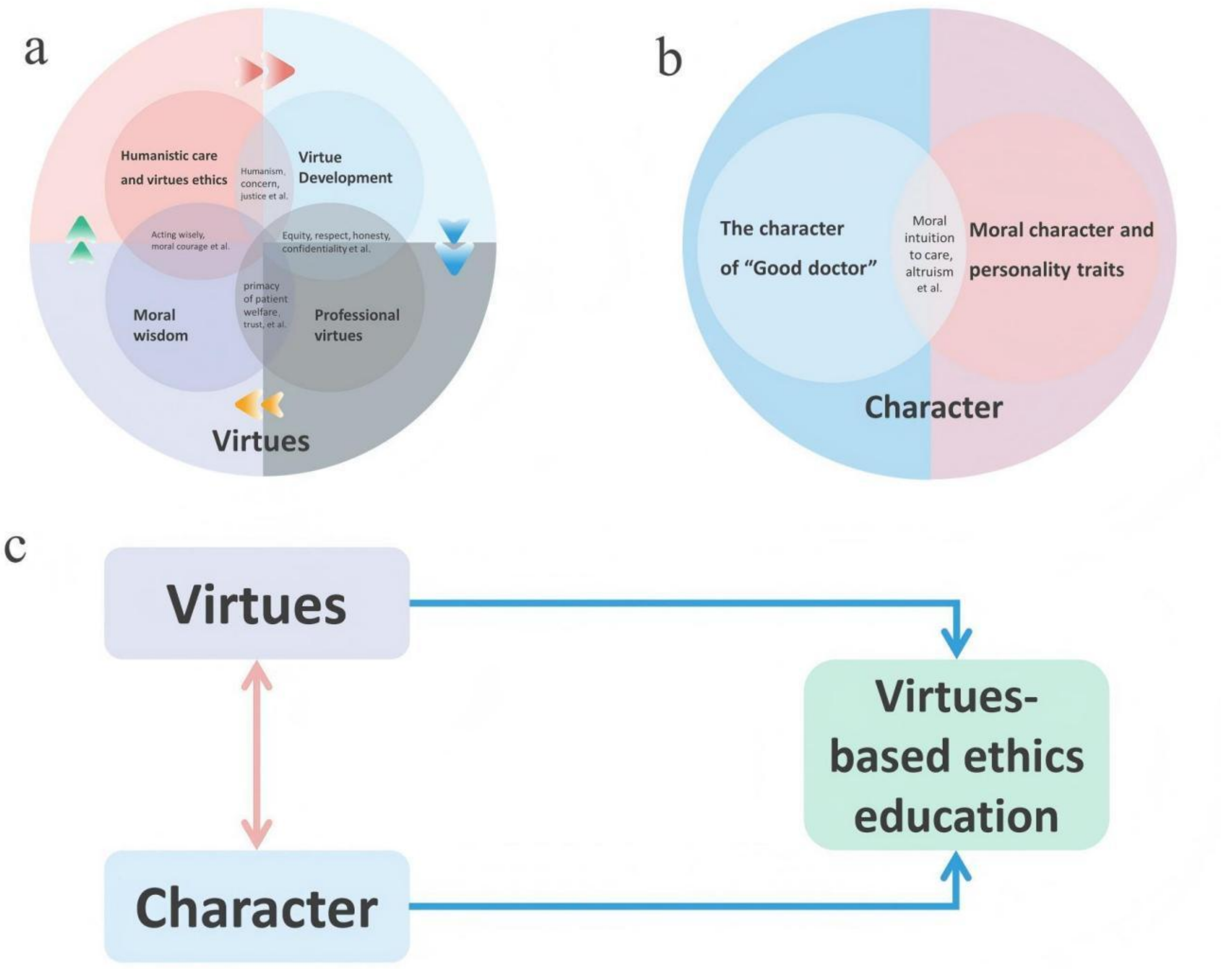

Figure 3 The framework of Inner Dimensions of Virtues and Characters
Note: Figure (a) indicates the four inner dimensions of virtues; figure (b) indicates the two inner dimensions of character; figure (c) indicates the relationship between virtues and character.

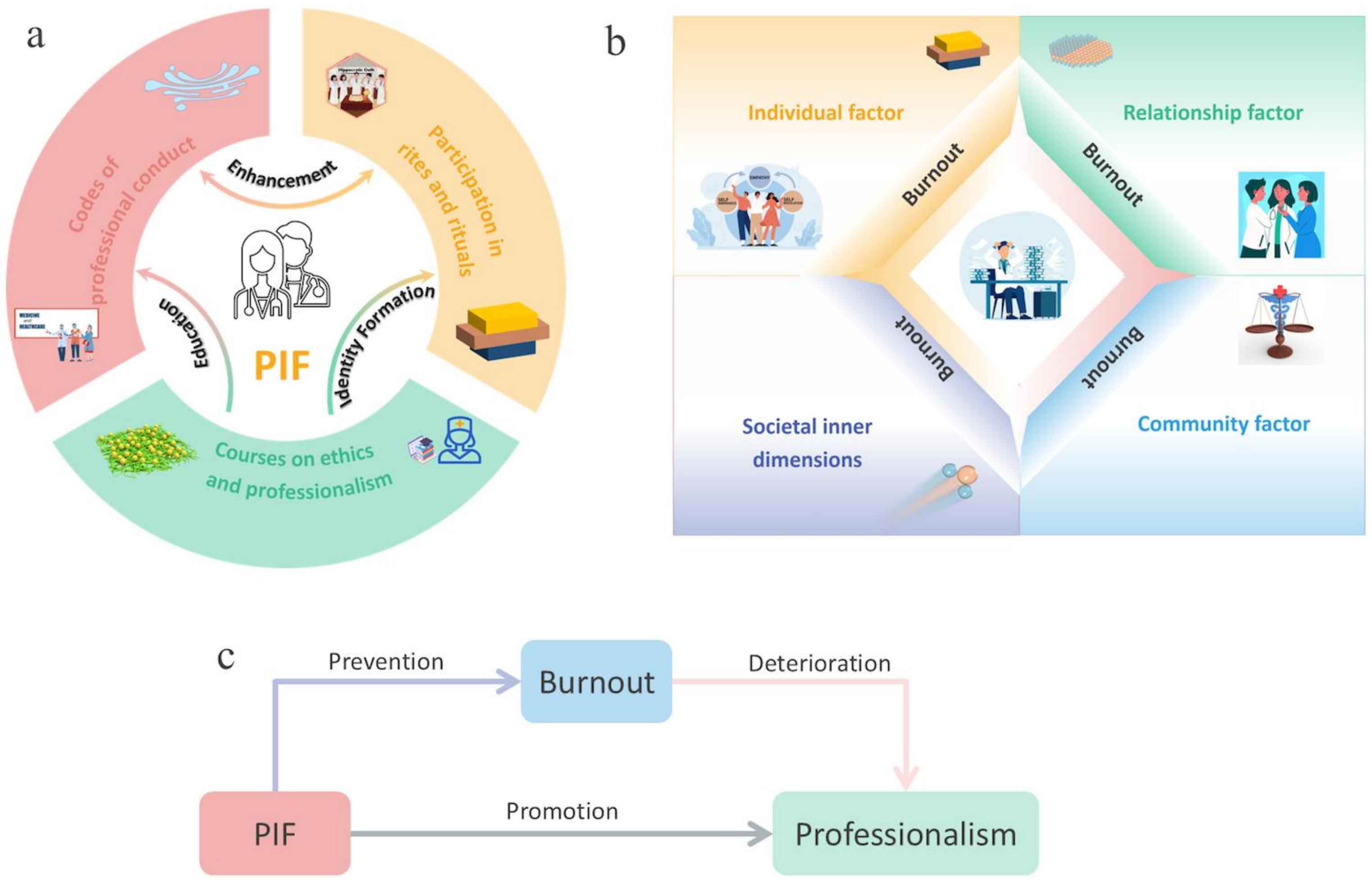

Figure 4 The framework of Inner Dimensions of Professionalism
Note: Figure (a) indicates the three inner dimensions of PIF; figure (b) indicates the four inner dimensions of burnout; figure (c) indicates the relationship between PIF and burnout.

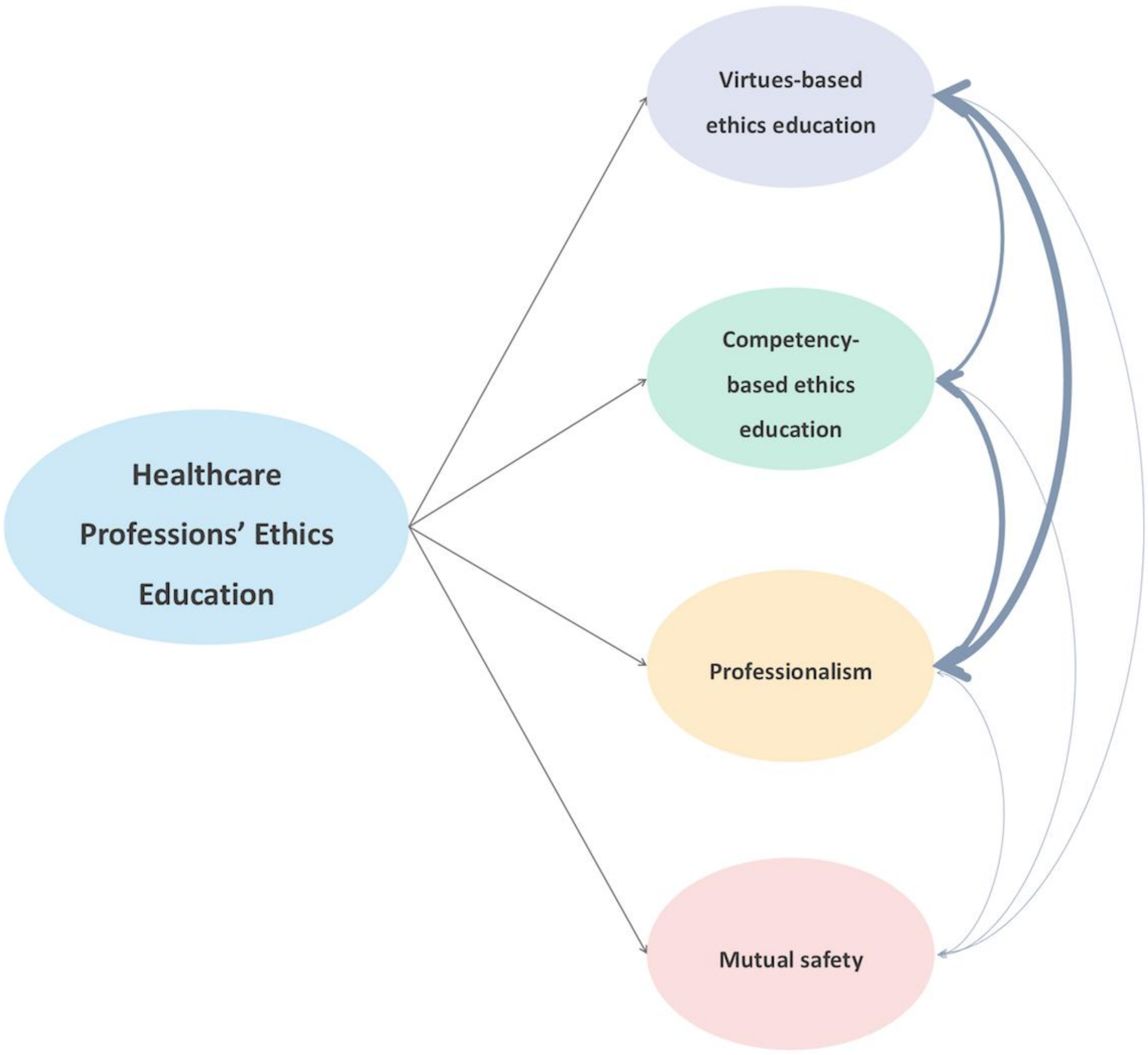

Figure 5 The relationship of four themes
Note: The thickness of the mutual arrow lines connecting the themes represents the closeness of the relationship and the frequency of interaction between them.

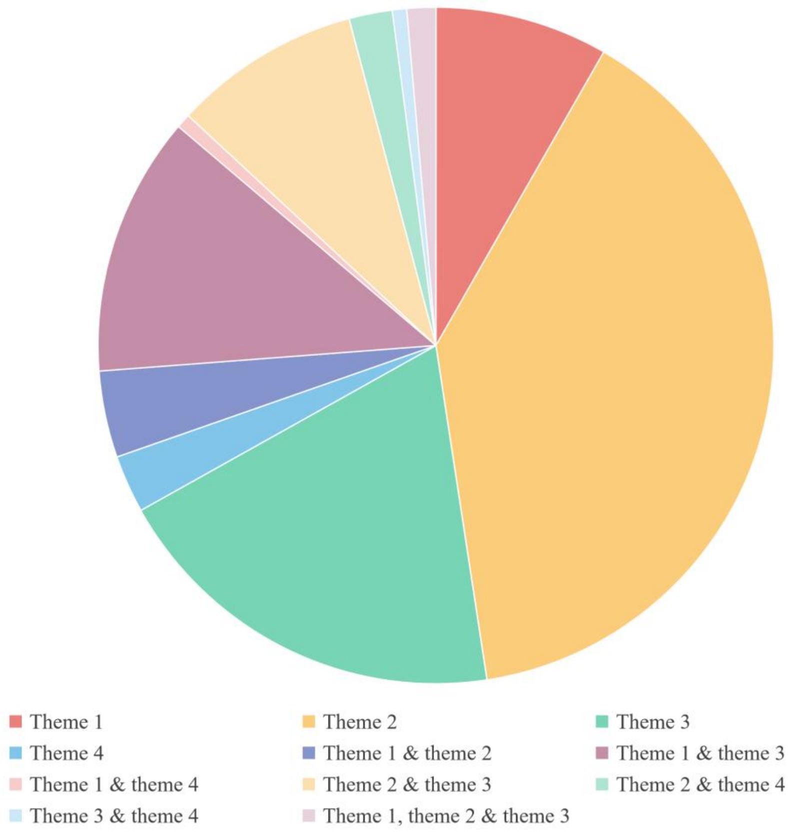

Figure S1 Pie chart of coding results

Notice: Theme 1 (N = 12); Theme 2 (N = 57); Theme 3 (N = 28); Theme 4 (N = 4); Theme 1 & theme 2 (N = 6); Theme 1 & theme 3(N = 18); Theme 1 & theme 4 (N = 1); Theme 2 & theme 3 (N = 13); Theme 2 & theme 4 (N = 3); Theme 3 & theme 4 (N = 1); Theme 1, theme 2 & theme 3 (N = 2). Theme 1, theme 2, theme 3, and theme 4 respectively represent virtues-based ethics education, competency-based ethics education, professionalism, and mutual safety.

Table S3 Preferred Reporting Items for Systematic reviews and Meta-Analyses extension for Scoping Reviews (PRISMA-ScR) Checklist

SECTION	ITEM	PRISMA-ScR CHECKLIST ITEM	REPORTED ON PAGE #
**TITLE**			
Title	1	Identify the report as a scoping review.	1
**ABSTRACT**			
Structured summary	2	Provide a structured summary that includes (as applicable): background, objectives, eligibility criteria, sources of evidence, charting methods, results, and conclusions that relate to the review questions and objectives.	1
**INTRODUCTION**			
Rationale	3	Describe the rationale for the review in the context of what is already known. Explain why the review questions/objectives lend themselves to a scoping review approach.	2-3
Objectives	4	Provide an explicit statement of the questions and objectives being addressed with reference to their key elements (e.g., population or participants, concepts, and context) or other relevant key elements used to conceptualize the review questions and/or objectives.	3
**METHODS**			
Protocol and registration	5	Indicate whether a review protocol exists; state if and where it can be accessed (e.g., a Web address); and if available, provide registration information, including the registration number.	Not registration
Eligibility criteria	6	Specify characteristics of the sources of evidence used as eligibility criteria (e.g., years considered, language, and publication status), and provide a rationale.	3
Information sources*	7	Describe all information sources in the search (e.g., databases with dates of coverage and contact with authors to identify additional sources), as well as the date the most recent search was executed.	3-4
Search	8	Present the full electronic search strategy for at least 1 database, including any limits used, such that it could be repeated.	3-4
Selection of sources of evidence†	9	State the process for selecting sources of evidence (i.e., screening and eligibility) included in the scoping review.	3-4
Data charting process‡	10	Describe the methods of charting data from the included sources of evidence (e.g., calibrated forms or forms that have been tested by the team before their use, and whether data charting was done independently or in duplicate) and any processes for obtaining and confirming data from investigators.	3-4,6
Data items	11	List and define all variables for which data were sought and any assumptions and simplifications made.	3-4
Critical appraisal of	12	If done, provide a rationale for conducting a critical appraisal	3-4

SECTION	ITEM	PRISMA-ScR CHECKLIST ITEM	REPORTED ON PAGE #
individual sources of evidence§		of included sources of evidence; describe the methods used and how this information was used in any data synthesis (if appropriate).	
Synthesis of results	13	Describe the methods of handling and summarizing the data that were charted.	5
**RESULTS**			
Selection of sources of evidence	14	Give numbers of sources of evidence screened, assessed for eligibility, and included in the review, with reasons for exclusions at each stage, ideally using a flow diagram.	5-17
Characteristics of sources of evidence	15	For each source of evidence, present characteristics for which data were charted and provide the citations.	5-17
Critical appraisal within sources of evidence	16	If done, present data on critical appraisal of included sources of evidence (see item 12).	5-17
Results of individual sources of evidence	17	For each included source of evidence, present the relevant data that were charted that relate to the review questions and objectives.	5-17
Synthesis of results	18	Summarize and/or present the charting results as they relate to the review questions and objectives.	5-17
**DISCUSSION**			
Summary of evidence	19	Summarize the main results (including an overview of concepts, themes, and types of evidence available), link to the review questions and objectives, and consider the relevance to key groups.	17-19
Limitations	20	Discuss the limitations of the scoping review process.	19
Conclusions	21	Provide a general interpretation of the results with respect to the review questions and objectives, as well as potential implications and/or next steps.	19-20
**FUNDING**			
Funding	22	Describe sources of funding for the included sources of evidence, as well as sources of funding for the scoping review. Describe the role of the funders of the scoping review.	20

JBI = Joanna Briggs Institute; PRISMA-ScR = Preferred Reporting Items for Systematic reviews and Meta-Analyses extension for Scoping Reviews.

* Where *sources of evidence* (see second footnote) are compiled from, such as bibliographic databases, social media platforms, and Web sites.

† A more inclusive/heterogeneous term used to account for the different types of evidence or data sources (e.g., quantitative and/or qualitative research, expert opinion, and policy documents) that may be eligible in a scoping review as opposed to only studies. This is not to be confused with *information sources* (see first footnote).

‡ The frameworks by Arksey and O'Malley (6) and Levac and colleagues (7) and the JBI guidance (4, 5) refer to the process of data extraction in a scoping review as data charting.

§ The process of systematically examining research evidence to assess its validity, results, and relevance before using it to inform a decision. This term is used for items 12 and 19 instead of "risk of bias" (which is more applicable to systematic

reviews of interventions) to include and acknowledge the various sources of evidence that may be used in a scoping review (e.g., quantitative and/or qualitative research, expert opinion, and policy document).

From: Tricco AC, Lillie E, Zarin W, O'Brien KK, Colquhoun H, Levac D, et al. PRISMA Extension for Scoping Reviews (PRISMAScR): Checklist and Explanation. Ann Intern Med. 2018;169:467–473. doi: 10.7326/M18-0850.
